# Management strategies for children with COVID-19: ESPR practical recommendations

**DOI:** 10.1007/s00247-020-04749-3

**Published:** 2020-07-03

**Authors:** Maria Raissaki, Susan C. Shelmerdine, Maria Beatrice Damasio, Seema Toso, Ola Kvist, Jovan Lovrenski, Franz Wolfgang Hirsch, Süreyya Burcu Görkem, Anne Paterson, Owen J. Arthurs, Andrea Rossi, Joost van Schuppen, Philippe Petit, Maria I. Argyropoulou, Amaka C. Offiah, Karen Rosendahl, Pablo Caro-Domínguez

**Affiliations:** 1 Department of Radiology, University Hospital of Heraklion, University of Crete, Crete, Greece; 2 Department of Clinical Radiology, Great Ormond Street Hospital for Children, London, WC1N 3JH UK; 3 UCL Great Ormond Street Institute of Child Health, Great Ormond Street Hospital for Children, London, UK; 4 NIHR Great Ormond Street Biomedical Research Centre, London, UK; 5 U.O.C. Radiologia, Istituto Giannina Gaslini, Genoa, Italy; 6 Department of Diagnostics, Geneva Children’s Hospitals, Geneva, Switzerland; 7 Department of Pediatric Radiology, Karolinska University Hospital, Stockholm, Sweden; 8 Faculty of Medicine, University of Novi Sad, Novi Sad, Serbia; 9 Institute for Children and Adolescent Health Care of Vojvodina, Novi Sad, Serbia; 10 Department of Pediatric Radiology, University of Leipzig, Leipzig, Germany; 11 Paediatric Radiology Department, Erciyes University School of Medicine, Children’s Hospital, Kayseri, Turkey; 12 Department of Radiology, Royal Belfast Hospital for Sick Children, Belfast, UK; 13 Neuroradiology Unit, Istituto Giannina Gaslini, Genoa, Italy; 14 Department of Health Sciences (DISSAL), University of Genoa, Genoa, Italy; 15 Department of Radiology and Nuclear Medicine, Amsterdam UMC, University of Amsterdam, Amsterdam, Netherlands; 16 Service d’imagerie pédiatrique et prénatale, Aix Marseille University, Hôpital de La Timone-Enfants, Marseille, France; 17 Department of Clinical Radiology and Imaging, Medical School, University Hospital of Ioannina, Ioannina, Greece; 18 Academic Unit of Child Health, University of Sheffield, Sheffield, UK; 19 Department of Radiology, Sheffield Children’s NHS Foundation Trust, Sheffield, UK; 20 Department of Radiology, University Hospital of North Norway, Tromsø, Norway; 21 Department of Clinical Medicine, UiT the Arctic University of Norway, Tromsø, Norway; 22 Unidad de Radiología Pediátrica, Servicio de Radiodiagnóstico, Hospital Universitario Virgen del Rocío, Sevilla, Spain

**Keywords:** Children, Coronavirus, COVID-19, Imaging protocol, Management, Radiology

## Abstract

During the outbreak of the COVID-19 pandemic, guidelines have been issued by international, national and local authorities to address management and the need for preparedness. Children with COVID-19 differ from adults in that they are less often and less severely affected. Additional precautions required in the management of children address their increased radiosensitivity, need for accompanying carers, and methods for dealing with children in a mixed adult-paediatric institution. In this guidance document, our aim is to define a pragmatic strategy for imaging children with an emphasis on proven or suspected COVID-19 cases. Children suspected of COVID-19 should not be imaged routinely. Imaging should be performed only when expected to alter patient management, depending on symptoms, preexisting conditions and clinical evolution. In order to prevent disease transmission, it is important to manage the inpatient caseload effectively by triaging children and carers outside the hospital, re-scheduling nonurgent elective procedures and managing symptomatic children and carers as COVID-19 positive until proven otherwise. Within the imaging department one should consider conducting portable examinations with COVID-19 machines or arranging dedicated COVID-19 paediatric imaging sessions and performing routine nasopharyngeal swab testing before imaging under general anaesthesia. Finally, regular personal hygiene, appropriate usage of personal protective equipment, awareness of which procedures are considered aerosol generating and information on how to best disinfect imaging machinery after examinations should be highlighted to all staff members.

**Electronic supplementary material:**

The online version of this article (10.1007/s00247-020-04749-3) contains supplementary material, which is available to authorized users.

## Introduction

Since the recent identification of a novel coronavirus (severe acute respiratory syndrome coronavirus 2, or SARS-CoV-2, the infection coronavirus disease 2019 [COVID-19]), there has been the declaration of a global pandemic, with a seemingly uncontrolled daily death toll, currently standing at more than 133,000 worldwide (as of 15 April 2020) [1]. There is an international state of fear, uncertainty and, for the most part, confusion. Confusion regarding best medical practices, confusion regarding population control and confusion regarding when and whether we will return to normality.

Understanding this new disease is indeed required. The scientific literature states that the SARS-CoV-2 virus is predominantly spread through human droplet transmission (i.e. via sneezing, coughing or close contact with others <2 m away) [2], with the greatest risk during the 2- to 14-day incubation period, when many individuals may be asymptomatic [2, 3]. Alternatively, the virus can remain viable for several days as aerosols or on hard surfaces [4] and fomite transmission is plausible if a person is in contact with an infected surface [4]. Children are susceptible to COVID-19, although they appear to have a less severe course of illness than adults [5] with only 3% suffering a severe illness and almost 13% being asymptomatic [6]. Only two paediatric deaths have been reported in the literature so far [6, 7], although more cases in all paediatric age groups have been described in news reports. Whilst transplacental and intrauterine transmission are not believed to occur, perinatal infection has been described via infected mothers [8, 9].

The literature regarding imaging of COVID-19 in children is predominantly based on small retrospective case series and reports, without clarification of the indications for imaging. Paradoxically, many infected cases may present with normal thoracic imaging [10] whilst those who have recovered may continue to display features of characteristic disease [11]. Given the importance of implementing measures to prevent further disease transmission and inappropriate medical treatment [12], our aims are to define a pragmatic strategy for imaging children in paediatric radiology departments with an emphasis on proven or suspected COVID-19 cases. We underscore some important learning points across different nations, and hope this will provide guidance and reassurance through better understanding and preparedness.

## General preparedness measures in hospitals and radiology departments

General preparedness for a pandemic requires involving the whole community, not only the health care system [13]. The aim is to reduce transmission both in public spaces and hospital settings, given that the main source of transmission is close contact between family members [14]. The National Health Commission of China reported that transmission between healthcare workers accounted for 3.8% of COVID-19 patients, lower than the respective reported incidences during the severe acute respiratory syndrome (SARS) and Middle East respiratory syndrome (MERS) coronavirus epidemics [15]. Several national authorities have therefore tried to minimize transmission through measures including countrywide curfews, closed borders, social distancing and self-quarantine.

The goal of radiology preparedness is to maintain the capacity for continued operation during these extraordinary times [12]. Solutions to reduce in-hospital transmission vary on local and national levels, and include, where feasible, a reduction in on-site staff and visitors to the hospital, maximizing the intensive care unit (ICU) capacity, managing patient flow and providing personal protective equipment measures.

The hospital entrance serves as the first point of physical contact where further access can be limited, and therefore patient triage is important [16, 17]. Paediatric wards in many countries have been divided into COVID-19 wards and ordinary (i.e. non-COVID-19) wards. Where possible, children who are being admitted or undergoing anaesthesia are prioritised for COVID-19 testing [18]. Entrance and exit routes for COVID-19 patients to various imaging areas should also be clearly identified.

Where imaging is needed, symptomatic children are treated as possible COVID-19 cases, especially in endemic areas. For urgent requests, portable imaging examinations (e.g., portable ultrasound or radiography) are preferred to avoid any transportation of potentially infected patients within the hospital and to reduce personnel exposure. Any healthcare professional at risk of interaction with potential COVID-19 patients should utilize personal protective equipment in accordance with local and national guidance, as well as adopt any protective covering for their machine to enable more thorough cleaning afterwards. Whilst this is the ideal situation, it is unfortunate that an unprecedented surge in demand for protective gear has meant that some regions are experiencing an acute shortage of supplies [19]; therefore, rational usage of equipment should be balanced with the need for protection. Change in policies should be explained by an infection control expert.

To reduce the number of on-site staff within hospitals, those equipped with teleradiology (i.e. remote working) facilities should adopt this as much as possible. On-call rotas should be modified with backup reserve colleagues ready to take the place of those with contact and obligatory quarantine or unexpected illnesses [12]. In many centres, all annual leave requests have been cancelled or reduced, depending on staffing levels and workload. Multidisciplinary team meetings should also be reduced to a minimum, and conducted via online communication software whenever possible. It is important to consider sensitive patient data and safety when using online meetings because some platforms have come under firm scrutiny [20]. Finally, personal hand hygiene and social distancing inside and outside the hospital is of utmost importance. Computer workstations, mobile and hospital phones, and hand-held dictation devices should all be disinfected multiple times a day and always between different staff usages. The use of shared devices such as photocopier machines and computers should be reduced.

## Clinical presentation of COVID-19 in the paediatric population

Common symptoms in children with COVID-19 include fever and cough (present in half of symptomatic cases), with fever usually subsiding within 3 days [7, 21]. Other symptoms include fatigue, myalgia, rhinorrhoea or gastrointestinal complaints (diarrhoea) [22]. Anecdotal evidence suggests that a loss of smell and taste occurs in a significant number of adults [23]; however, it is uncertain if this occurs in children and it has not been recognized by the World Health Organization (WHO) as a formal symptom of COVID-19 [24]. Maternal hypoxaemia from COVID-19 may cause intrauterine asphyxia, leading to premature delivery in 47% of cases in one study, as well as stillbirth [25]. In another study, this one of 33 neonates born to infected mothers, only 3/33 (9%) were infected, suffered mild tachypnoea [26] and fully recovered within 6 days. Laboratory abnormalities vary while a consistent pattern of derangements is not found in children [27]. Leucopenia is relatively common, being documented in approximately 30% of children [28]. Elevated procalcitonin levels are reportedly suggestive of bacterial co-infection, and raised C-reactive protein (CRP) occurs with severe infection [27]. Elevation of D-Dimer levels is less commonly encountered in children compared to in adults [29].

There is little evidence of outcomes in children with coexisting health problems, although these are known to be associated with poor outcomes in children who have suffered from previous strains of coronavirus [30], and in adults with COVID-19 [31]. The latest guidance from the Royal College of Paediatrics and Child Health [18] highlights four categories of children at risk, where a lower threshold for investigation and treatment should be considered. These include: those with long-term respiratory conditions (e.g., asthma, chronic lung disease of prematurity), immunocompromised status (e.g., congenital immunodeficiency, post-transplant, immunosuppressive medication), haemodynamically significant or cyanotic heart disease and chronic kidney disease (stages 4 or 5 or on dialysis).

Due to the recent outbreak of this disease, long-term follow-up data are lacking. Different guidelines appear to grade the severity of COVID-19 in children according to different criteria (Online Supplementary Material Table S1) [16, 22, 32, 33], and there is still a lack of information regarding how well these categories predict clinical outcomes. In a large cohort from Wuhan, China, 149/171 (87.1%) of children [7] completely recovered, with only 3/171 (1.8%) requiring intensive care unit admission.

Regarding complications in paediatric COVID-19, childhood deaths are rare [6, 7]. Intussusception has been reported [7], although whether this is specific to COVID-19 or a result of other underlying viral infection remains to be seen. Lymphadenopathy, cavitation and lung abscesses have not been reported, and pleural effusions are unusual. Coinfection with other bacterial or viral organisms is well documented [34] and could potentially lead to complications such as cardiac dysfunction, liver damage and renal failure [35].

## Triage: assessment and prioritization

In general, patient assessment is recommended as per the WHO definition by inquiring about fever, symptoms of respiratory tract infection and/or close contact with someone with confirmed or suspected COVID-19 [36]. Ideally paediatric triage should be performed outside the hospital either via phone (through help desk lines [37]) or in dedicated COVID-19 areas within the emergency or outpatient departments [37, 38].

Admitting patients from the emergency department to the wards is based upon clinical status and preexisting conditions [39]. Clinical pathways that aim to keep children out of the hospital as much as possible should be adopted for asymptomatic and mild cases, with strict instructions for quarantine and observation at home [38], with the possibility of a rapid return upon clinical deterioration [39]. In this context, in European paediatric hospitals, triage of children is routinely based on clinical factors rather than imaging [39]. This contrasts with recommendations from China and Iran where imaging is a routine part of the triage in the hope of identifying abnormalities when rRT-PCR (real-time reverse transcriptase polymerase chain reaction) testing is negative or unavailable [11, 40, 41]. Once the child is admitted to the hospital, clear documentation of COVID-19 status is paramount. All charts, both electronic and paper (depending on the setting), should carry a warning status about COVID-19 testing. All radiology requests should mention the infection status of the patient.

When possible, patients should be scheduled appropriately to avoid overlap and contact in waiting areas, and additional time should be given to enable proper cleaning of machinery. People accompanying the child (limited to one in most centres) should also be screened for any relevant symptoms or possible contact with COVID-19 patients in the preceding 14 days, and if present, the appointment should be rescheduled or performed by personnel in the appropriate personal protective equipment (i.e. managed as if COVID-19 positive). In some European institutions, a negative PCR test is strongly recommended for any nonurgent imaging under general anaesthesia, and performed 2 days before the study.

## Indications for imaging children with COVID-19

There are no internationally agreed-upon guidelines for imaging children with COVID-19. Clinical decision pathways for the diagnosis, management and treatment for COVID-19 have varied across different countries, and also across different institutions within the same country.

In general, rRT-PCR of the viral RNA, performed on nasopharyngeal swab testing, remains the reference standard for diagnosis [42]. Nevertheless, this test may suffer from limited sensitivity rates (of approximately 70%), short supply and can take time to process (1–3 days) [43]. Due to these constraints, chest CT has been used in adults as a tool for diagnosis [44]; however, approximately 16% of children with COVID-19 exhibit no radiographic findings, and in 7% there are imaging findings of pneumonia but no symptoms [7]. We therefore support the recommendations by the Royal College of Paediatrics and Child Health [18] and Italian Society of Medical and Interventional Radiology [45], which strongly advise that imaging should not be used routinely for the diagnosis of COVID-19 in children. Findings are typically nonspecific for SARS-CoV-2, and include patchy consolidation and ground-glass opacification, which may mimic other viral pathogens [46]. All imaging should be reserved for selected cases where results are anticipated to alter the child’s management. Non-thoracic imaging (any modality) should be discussed on a case-by-case basis and, if not urgent, should be performed at a later stage.

**Fig. 1:**
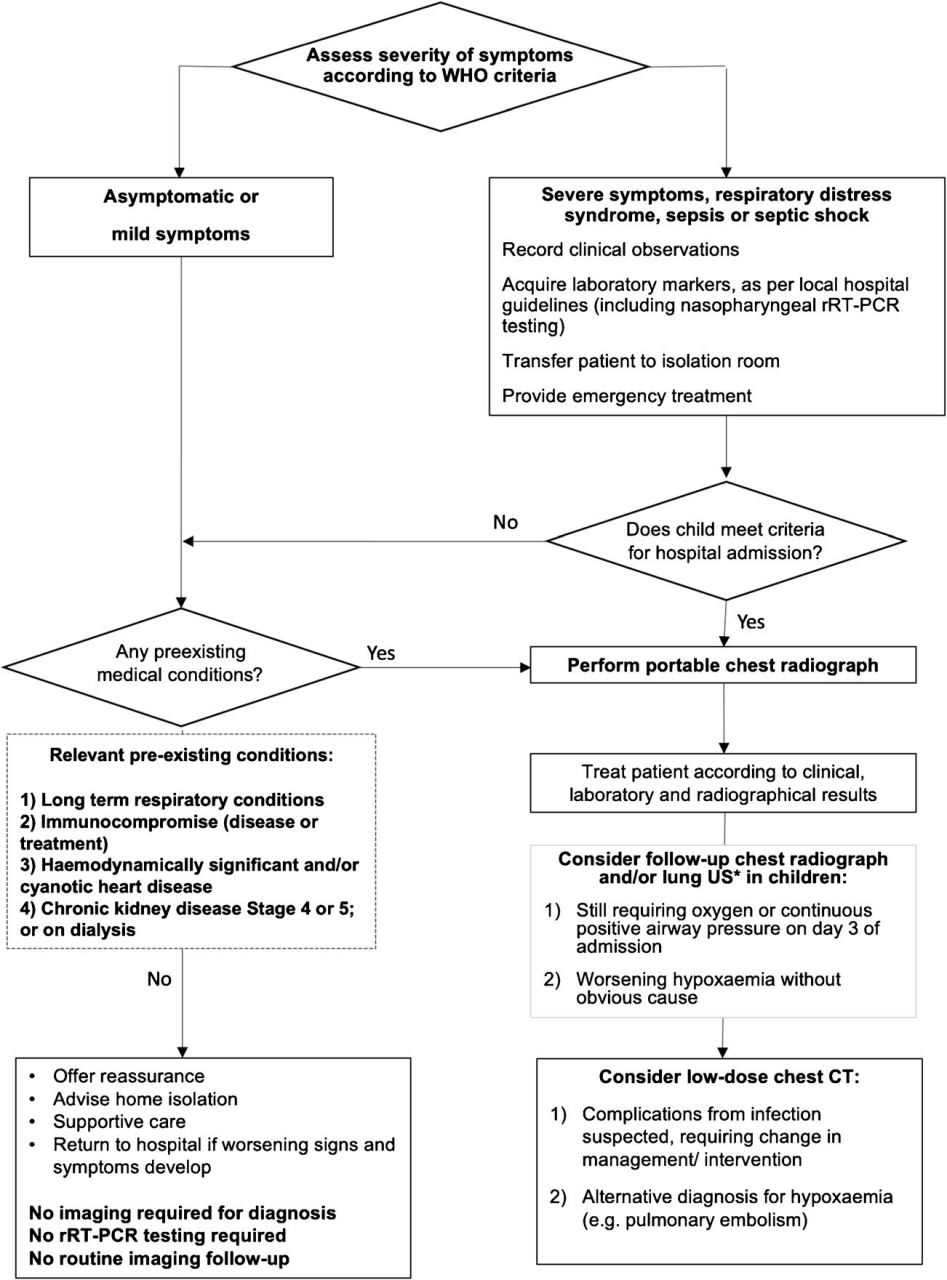
Imaging pathway for a child with suspected COVID-19 related to clinical presentation. This pathway is meant as a resource and was generated based on modification of available European guidelines. Each child may require individual case-by-case discussion with the radiologist. Severity scoring according to WHO guidelines [33] is provided in Online Supplementary Material Table S1. Mild symptoms are defined as those of pneumonia, without the requirement for supplementary oxygen. Relevant preexisting conditions and guidance regarding follow-up chest radiographs are taken from Royal College of Paediatrics and Child Health in the United Kingdom and British Paediatric Respiratory Society guidelines [18, 39]. * Only portable and by experienced operators. *rRT-PCR* real time reverse transcriptase polymerase chain reaction*, WHO* World Health Organization

With this in mind, a pragmatic imaging pathway has been included for guidance. This considers the severity of symptoms according to the WHO guidelines [36], preexisting health conditions [18] and attempts to minimize imaging exposure and rRT-PCR testing for asymptomatic and milder cases, thereby preserving resources and radiologic personnel exposure (Fig. 1).

Specific guidances for chest imaging in suspected/confirmed COVID-19 cases are provided below.

### Radiography

Chest radiographs can be performed as a first-line tool in symptomatic children with need for hospitalization or underlying health conditions, and to exclude other causes for respiratory complaints. Use of radiographs as a follow-up for children with a deteriorating condition to assess complications is also recommended. Lateral chest radiographs are not indicated.

### CT

There is no routine indication for performing chest CT in children with COVID-19. A CT scan with a dose appropriate for the child should be performed only when required to answer a specific clinical query. Special consideration should be given where ICU admission or urgent intervention/surgery is required, in children with clinical deterioration despite therapy, for underlying health conditions or to exclude other causes of respiratory distress (e.g., pulmonary embolism). In many published articles, chest CT is mostly performed without contrast, although the administration of intravenous iodinated contrast medium is preferred in suspected pulmonary embolism or when there is a question for mediastinal abnormality.

### Ultrasound

There are no guidelines that advocate routine usage of lung US in children with COVID-19. Reports from Italy have advocated chest US in the ICU as a monitoring tool to detect lung collapse and consolidation to aid ventilator settings or patient positioning (from supine to prone) [47, 48]. This practice requires experience with lung US and while it may reduce the number of repeated portable radiographs performed, introducing such a service would depend on local expertise, the availability of portable machine and staffing. US may play a role as a follow-up tool and may be requested to exclude deep venous thrombosis in selected patients due to the known thrombotic effect of COVID-19.

### MRI

The use of MRI for the initial diagnosis of COVID-19 infection or in the further course of the disease has not been reported and is therefore not recommended.

### Follow-up imaging

Repeat chest imaging is not required as standard routine practice, and should not be performed in children with improving clinical condition and when results are not expected to alter clinical management.

## Standardized reporting of chest CT: current role in children

There are no mandatory European guidelines in place for structured CT reporting of COVID-19 in children. Nevertheless, preexisting guidelines (developed predominantly with adults in mind) could potentially provide a useful structure to collate information for future imaging research.

The Radiologic Society of North America (RSNA) recently proposed a reporting language relevant to specific CT appearances [49], categorized into four classes (typical, intermediate, atypical appearances and negative for pneumonia), taking into account the potential of overlap with other diseases (Online Supplementary Material Table S2). The Dutch Society of Radiologists has proposed a reporting system entitled “CO-RADS” [50]. In this, the level of suspicion is graded from CO-RADS 1 to CO-RADS 6 (Online Supplementary Material Table S3) with underlying comorbidities and differential diagnoses considered. Finally, Li et al. [51] have developed a CT total severity score for adults with COVID-19 (Online Supplementary Material Table S4), taking into account the extent of inflammatory lesions for each lobe, and scored from 0 (0%) to 4 (76–100%). The total sum of scores in all lobes provides a total severity score range of 0–20, which the authors suggest could reflect the clinical severity and therefore patient outcome. One should bear in mind that these systems have not been tested in children in whom CT findings may be absent or mild [6, 7, 11] and can overlap with other (viral) infectious diseases [52]. Therefore, care should be taken when adopting these tools for disease probability or for clinical prognosis.

## Considerations for personal protective equipment in paediatric radiology departments

Appropriate personal protective equipment prevents expected COVID-19 transmission through droplet and contact transfer, and also inhibits aerosol transmission where aerosol-generating procedures are conducted [53]. The latter mostly include procedures carried out in the ICU, or emergency department or theatre (e.g., intubation, extubation, bronchoscopy, dental procedures, continuous positive airway pressure [54]) and are less common in radiology departments [54]. Hence, the routine use of a particulate respirator mask (FFP) as an airborne precaution is not usually necessary in radiology [19] unless urgent imaging is required whilst an aerosol-generating procedure is simultaneously occurring in the vicinity (e.g., during portable studies). The Society for Interventional Radiology has produced a list of aerosol-generating procedures and propose a pathway for resource allocation [55]. It should be borne in mind that any procedure in which the child becomes distressed is potentially an aerosol-generating procedure and different departments will have their own recommendations. In many European centres, intussusception reduction is considered an aerosol-generating procedure and is performed with water/contrast and not air, by personnel in appropriate personal protective equipment, and preferably in an operating theatre where better ventilation conditions exist. Contact and droplet precautions are generally well addressed in paediatric radiology departments while airborne precautions are harder to implement because negative-pressure rooms are not readily available. In these cases, a local exhaust ventilation device could be considered, but may be hard to obtain [56, 57]. The continued use of positive-pressure rooms to protect the patient versus negative-pressure rooms is also controversial [56].

Children with suspected or confirmed COVID-19 requiring imaging either as a portable study or in the radiology department (e.g., CT, MRI, fluoroscopy) should wear a surgical mask. This may prove challenging for toddlers and babies, where exceptions may often be made. In some centres, children will wear an additional protective gown and/or gloves. Non-COVID hospitalized children are not required to wear masks; however, accompanying adults in most European hospitals should be encouraged to wear a surgical mask. For radiation-based procedures, the accompanying adult is provided with a lead apron that should remain within the imaging room and be wiped down along with all other apparatus after the procedure. All other lead aprons should be stored in areas that do not face patients.

For radiology personnel, protection varies depending on the procedure. Table 1 was generated based on modified guidelines from numerous international organizations [19, 58–62]. It should be noted that within each paediatric radiology department, there are slightly modified procedures based on the local level of risk, resources and hospital-wide/nationwide guidelines. Many departments suggest that all staff in direct contact with patients wear hospital uniforms/scrubs that they change into upon arriving at work. For routine paediatric radiology daily practice, recommendations vary from surgical masks and handwashing to plastic aprons, gloves and standard surgical masks.

**Table 1:** Rational use of personal protective equipment for coronavirus disease COVID-19

Setting	Target personnel or patients	Activity	Type of precautions	Type of PPE or procedure
Patient room	Radiologist/radiographer	Providing direct care to a COVID-19 child	Contact and droplet	Surgical mask Gown Gloves Eye protection
		Present while aerosol-generating procedures are performed on COVID-19 patient	Contact, droplet and aerosol	Attempt delaying imaging If not possible: respirator maskN95/FFP2/FFP3^b^, gown, gloves and eye protection
Radiology consulting room	Radiologist/radiographer/ sonographer	Examination of patient with respiratory symptoms	Contact and droplet	Surgical mask Gown Gloves Eye protection
		Examination of patient without respiratory symptoms	Contact and droplet	Surgical mask Gown/scrubs Hand hygiene/gloves Eye protection
	Accompanying carer/chaperone	Accompanying child to consultation. No direct contact with medical staff/child during consultation	Contact and droplet	Surgical mask Hand hygiene
Radiology waiting area	Patients and carers with/without respiratory symptoms	Waiting for consultation	Contact and droplet	Limit number of people Surgical mask, if tolerated(difficult in young children)
Radiology administrative areas	All staff	Administrative tasks	None	Maintain spatial distance of 1 m Surgical mask
Fluoroscopyprocedures^a^	Radiologist/radiographer/ nurse	Voiding cystourethrogram, barium enema	Contact and droplet	Limit number of people Delay non-urgent cases Surgical mask Gown Gloves Eye protection
		Swallowing study	Contact, droplet and aerosol	Limit number of people Delay non-urgent cases Respirator mask (N95/FFP2/FFP3) ^b^ Gown Gloves Eye protection

*PPE* personal protective equipment

^a^Fluoroscopy procedures are not outlined by specific guidelines. Voiding cystourethrogram and barium enema guidelines were based on those for caregivers in contact with urine and faeces while treating COVID-19 positive patients. The suggestions for swallowing studies are based on available information from aerosol-generating procedures such as upper gastrointestinal endoscopy or dental procedures, though this remains debatable

^b^As per national guidelines

Health care workers should wear a mask (either surgical or respirator N95, FFP2 or FFP3), gown, gloves and eye protection when working with children with suspected or confirmed COVID-19. For aerosol-generating procedures, a respirator mask and a waterproof apron should be added [19]. In the United Kingdom, an FFP3 mask is preferred. It is recommended that all staff are fitted for all different makes of masks used in their institution to ensure that an acceptable seal is achieved. Lead aprons should be worn beneath personal protective equipment, which should include a full-length, long-sleeve, water-resistant apron over the lead for aerosol-generating procedures, whereas standard plastic aprons are sufficient for non-aerosol-generating procedures. Both mentioned practices may vary according to local regulations and resources. For urgent fluoroscopy procedures in children with suspected COVID-19 that cannot be delayed, surgical masks, gown, gloves and eye protection should be used [19]. For urgent upper gastrointestinal contrast studies, we suggest an FFP2/FFP3/N95 mask, gown, gloves and eye protection. Although this procedure may not generally be considered aerosol generating, it can be considered as such should suction be required. Administrative staff do not need to wear personal protective equipment, although many are choosing to wear surgical masks as a precaution.

### Donning and doffing personal protective equipment

Instructional resources for putting on (donning) and taking off (doffing) personal protective equipment are provided by public health authorities [63] and the Centres for Disease Control and Prevention [64]. Some centres/authorities have produced documents and videos explaining donning and doffing for aerosol-generating [65] and non-aerosol-generating procedures [66].

In brief, for donning, these include hand hygiene, followed by gown, mask, eye protection and finally gloves. For doffing, these include removing gloves, eye protection, gown and finally the mask without touching the outside of the protective equipment. In some departments, glove removal is followed by hand hygiene, with new gloves worn before gown removal, then hand hygiene, eye protection removal and finally removing the mask [63]. Posters or notices describing the sequences should be placed in clinical areas where donning and doffing are routinely expected to occur in appropriately labelled no-touch bins.

### Radiographer teams for working with children with COVID-19

Common practice amongst radiographers in Europe include having a person or team assigned as warm/dirty/contaminated, who comes into relative close proximity with the patient, and another assigned as cold/clean to carry out portable radiography and/or to operate the CT and fluoroscopy machines. The warm/dirty/contaminated individual wears a lead apron and full personal protective equipment, and positions the patient in the CT room/fluoroscopy couch, whereas the cold/clean individual wears a surgical mask, apron and gloves (for portable imaging) or remains in the CT control room/behind the screen in the fluoroscopy room, without any additional protection. The cold/clean individual acquires the images and has no patient interaction.

## Planned elective care for children without COVID-19

Clinicians in many countries have been instructed to postpone routine clinical work [67–70], including postponing radiology appointments across all modalities; in Northern Ireland for example, the deferral period has initially been defined as 2–3 months [68]. Reducing the number of children and their carers in radiology departments will both lessen the risks of cross-infection between staff and patients (and vice versa) and protect the most vulnerable with suppressed immune systems and with complex needs. Urgent and emergent radiology exams will take place in a timely fashion as normal.

Ongoing communication between radiology consultants and their clinical colleagues is imperative to ensure there is a shared definition of what constitutes a routine case; patient care pathways may need to be adjusted to incorporate a delay in screening radiology examinations, for example neonates with a suspected genetic syndrome. Some US examinations in paediatric radiology are urgent simply because of skeletal maturation over a matter of time: hip US for suspected developmental dysplasia, head US whilst the anterior fontanelle remains large enough, and spine US before the posterior vertebral elements ossify and obscure the field of view. Multidisciplinary team decisions are required to answer questions regarding how long an individual child’s study can be postponed. Electronic access to patient clinical records is helpful to radiologists when vetting request forms to prioritise which studies must be performed.

Over time, it will be necessary to revisit the original requests for those postponed studies as they cannot be held off indefinitely. Discussion of patient needs with our colleagues must be ongoing. Collaborative electronic communication platforms and phone calls should be utilised.

## Special considerations in mixed adult-paediatric settings

Children being cared for in mixed adult-paediatric environments may be in contact with a greater number of COVID-19 patients and be imaged using the same equipment. Strict cleaning regimes should be adhered to and, where possible, dedicated paediatric lists and equipment should be employed. Local dedicated paediatric radiologists or adult radiologists familiar with paediatric imaging should continue to ensure children are imaged according to accepted protocols and guidelines.

With an increasing number of infected adults requiring inpatient admission, it is likely that some hospitals will need to convert some or all of their paediatric inpatient ward and intensive care beds for adults. It would be prudent to have contingency plans to close smaller paediatric units to centre care within tertiary units. Some staff may be required to temporarily relocate or utilise picture archiving and communication system (PACS) networks to report studies from other centres in the event of staff isolation or sickness [71].

## Equipment management and disinfection procedures

Where a suspected or confirmed COVID-19 patient requires imaging, this is preferentially performed as a portable study; if this is not possible, such as for CT or MRI, then a designated COVID-19 machine (if resources allow) should be assigned to avoid disease transmission between different machines. If there is only one CT and/or one MRI machine on site, dedicated rotations of COVID-19 positive/COVID-19 negative patients, might be required.

### CT/MRI

Before any imaging in the department, all unnecessary equipment and toys should be removed. Where an infected child is due to attend the department, the waiting room should be emptied and any central ventilation of the room (e.g., air conditioning) should be turned off. If performing an MRI study, ventilation in the gantry should be increased to the maximum level.

Where possible, imaging couches and beds should be covered with plastic drapes. After imaging examinations are performed, the room should be left for at least 20 min with the door closed to allow aerosol droplets to settle. According to American College of Radiology guidelines, depending on air exchange rates, the room could be out of service for approximately 1 h after imaging an infected patient [42]. Disinfection procedures should be performed according to local guidelines; however, viricidal agents should be used and viruses can be inactivated using lipid solvents such as ether, 75% ethanol, chlorine-containing disinfectant, peracetic acid and chloroform [72]. All surfaces, including doorknobs and equipment, should be cleaned. In the MRI rooms, the coils and gantry should also be wiped.

Floors should be cleaned and any windows in the waiting areas should be opened to allow natural ventilation. It has been reported that COVID-19 is sensitive to ultraviolet light and heat [72] and if a department has access to ultraviolet lamps for sterilization, this could be useful to implement in the imaging rooms. Mobile ultraviolet lamps can be used for 30–60 min at a time several times per day in between appointments when a room is empty.

### Ultrasound

Ultrasound imaging in children with COVID-19 will be performed as a portable study when possible. Before imaging, all unnecessary accessories/probes should be removed from the machine. In some centres, keyboard and probe covers (or plastic gloves) are also applied. After the examination, the US machine should be thoroughly cleaned before leaving the isolation room and again after exiting the isolated area. Probes should be cleaned by wiping the gel from the transducer followed by disinfection [73], using surface disinfectant wipes according to American Institute of Ultrasound in Medicine guidelines [74]. This should include the use of viricidal agents [75]. The entire probe (including the cables), the US screen, keyboard, transducer gel bottle and surfaces visible to the air and within 1 m from the patient should be disinfected.

### Interventional radiology

Any urgent interventional procedures should, depending on the age of the patient, be performed under local anaesthesia by the patient’s bedside when possible. Minor interventions in older children can be performed using a local anaesthetic. If sedation is required, the procedure should take place in the operating rooms under isolation restrictions, or alternatively, if resources allow, in a designated COVID-19 interventional room.

## Conclusion

Measures to prevent disease transmission during the COVID-19 pandemic within paediatric radiology departments and in the course of radiologic procedures are of paramount importance. Paediatric radiology staff can make an appreciable difference by adhering to pragmatic guidelines for imaging children with suspected or confirmed COVID-19. Whilst practices are mostly homogeneous across Europe, those of individual institutions may vary depending on the local catchment population, availability and expertise of personnel, equipment and resources. We have provided advice to professionals who care for children in radiology departments, suitable for adaptation in different settings and hope that this approach will be useful in the fight against this outbreak.

## Electronic supplementary material

ESM 1 (DOCX 24 kb)
