# Supplementary material for: Management strategies for children with COVID-19: ESPR practical recommendations
Source: Pediatr Radiol. 2020 Jul 3;50(9):1313–23. doi: 10.1007/s00247-020-04749-3 (PMC7332738; doi:10.1007/s00247-020-04749-3)
Supplement: Supplementary file 1 — (DOCX 24 kb) [file 247_2020_4749_MOESM1_ESM.docx]

**Electronic Supplementary Material**

**Table S1:** Differences in severity scoring for COVID-19 in children, between different published guidelines. Adapted from Cheng ZJ et al, 2020 [22]

*ARI acute respiratory infection, BP blood pressure, bpm beats/minute, CPAP continuous positive airway pressure, FiO2 fraction of inspired oxygen, MAP mean arterial pressure, NIV non-invasive ventilation, SBP systolic blood pressure, SD standard deviation, SIRS systemic inflammatory response syndrome, SpO2 oxygen saturation, ARDS acute respiratory distress syndrome, HR heart rate, PaO2 partial press of oxygen, SBP systolic blood pressure, ICU intensive-care unit, MODS multiple organ dysfunction syndrome, OI Oxygenation Index, OSI Oxygenation Index*

| **WHO guideline [34]** | **Definition for children**  **(2 months – 5 years old); otherwise adult criteria apply** | **Chinese 4^th^ edition guideline [22, 32]** | **Generic Definition** | **Chinese Expert Consensus Statement, Feb 2020 [33]** | **Generic Definition** |
| --- | --- | --- | --- | --- | --- |
| - | - | - | - | Asymptomatic | No clinical symptoms.  Normal chest imaging. |
| Mild &  Non-Severe Pneumonia | Uncomplicated URTI, with non-specific symptoms of fever, fatigue cough, anorexia, malaise, muscle pain, sore throat, dyspnoea, nasal congestion or headache.  Child with non-severe pneumonia has cough or difficulty breathing + fast breathing: fast breathing (in breaths/ min):  < 2 months, ≥ 60;  2–11 months, ≥ 50;  1–5 years, ≥ 40 and no signs of severe pneumonia | Mild | Patient presents with common symptoms: fever, dry cough, fatigue, headache,  sore throat | Mild | Symptoms of acute upper respiratory tract infection, including fever, fatigue, myalgia, cough, sore throat, runny nose, and sneezing.  Physical examination shows congestion of the pharynx and no auscultory abnormalities. |
| Severe | Child with cough or difficulty in breathing, plus at least one of the following:  1. Central cyanosis or SpO2 < 90%  2. Severe respiratory distress  3. Signs of pneumonia with a general danger sign: inability to breastfeed or drink, lethargy or unconsciousness, or convulsions  4. Other signs of pneumonia may be present: chest in-drawing, fast breathing (in breaths/min):  < 2 months, ≥ 60  2–11 months, ≥ 50  1–5 years, ≥ 40 | Severe | Patient fits any one of the following conditions:  1. Respiratory rate ≥ 30 breaths/ min  2. SpO2 ≤ 93%  3. PaO2/ FiO2 ≤ 300 mmHg  (1 mmHg = 0.133 kPa) | Moderate | Pneumonia, fever and mostly dry cough, followed by productive cough.  Some may have wheezing, but no obvious hypoxemia such as shortness of breath, and lungs can hear sputum or dry snoring and / or wet snoring. |
| Acute respiratory distress | 1. Onset: new or worsening respiratory symptoms within one week of known clinical insult  2. Chest imaging (radiograph, CT scan, or lung ultrasound): bilateral opacities, not fully explained by effusions, lobar or lung collapse, or nodules  3. Origin of oedema: respiratory failure not fully explained by cardiac failure or fluid overload.  2. Bilevel NIV or CPAP ≥ 5 cmH2O via full face mask: PaO2/  FiO2 ≤ 300 mmHg or SpO2/ FiO2 ≤ 264,  Mild ARDS (invasively ventilated): 4 ≤ OI < 8 or 5 ≤ OSI < 7.5  Moderate ARDS (invasively ventilated):  8 ≤ OI < 16 or 7.5 ≤ OSI < 12.3  Severe ARDS (invasively ventilated):  OI ≥ 16 or OSI ≥ 12.3 | Life-threatening | Patient fits any of the following conditions:  1. Patient presents with respiratory distress and needs mechanical ventilation support  2. Patient presents with shock  3. Patient presents with MODS and requires ICU admission. | Severe | Early respiratory symptoms such as fever and cough, may be accompanied by gastrointestinal symptoms such as diarrhoea.  Disease progression around 1 week, with dyspnoea, central cyanosis. Oxygen saturation is less than 92%, with other hypoxia manifestations. |
| Sepsis | Children: suspected or proven infection and ≥ 2 SIRS criteria, of which one must be abnormal temperature or white blood cell count |  |  | Critical | Progression to acute respiratory distress syndrome (ARDS) or respiratory failure, and may also have shock, encephalopathy, myocardial injury or heart failure, coagulation dysfunction, and acute kidney injury. Organ dysfunction can be life threatening. |
| Septic Shock | Patients presents any hypotension (SBP < 5th centile or > 2 SD below normal for age) or 2–3 of the following:  1. Altered mental state  2. Tachycardia or bradycardia (HR  < 90 bpm or > 160 bpm in infants  and HR < 70 bpm or > 150 bpm in  children)  3. Prolonged capillary refill (> 2 s) or warm vasodilation with bounding pulses; tachypnoea  4. Mottled skin or petechial or purpuric rash; increased lactate  5. Oliguria; hyperthermia or hypothermia |  |  |  |  |

**Table S2:** Imaging classification, rationale with CT findings and suggested reporting language per category [50]. *GGO – ground glass opacification*

| **COVID-19 pneumonia imaging classification** | **Rationale** | **CT Findings** | **Suggested Reporting Language** |
| --- | --- | --- | --- |
| Typical appearance | Commonly reported imaging features of greater specificity for COVID-19 pneumonia. | Peripheral, bilateral, GGO* with or without consolidation or visible intralobular lines (“crazy-paving”)  Multifocal GGO of rounded morphology with or without consolidation or visible intralobular lines (“crazy-paving”)  Reverse halo sign or other findings of organizing pneumonia (seen later in the disease) | “Commonly reported imaging features of (COVID-19) pneumonia are present. Other processes such as influenza pneumonia and organizing pneumonia, as can be seen with drug toxicity and connective tissue disease, can cause a similar imaging pattern.” |
| Indeterminate appearance | Nonspecific imaging features of COVID-19 pneumonia. | Absence of typical features AND  Presence of:  Multifocal, diffuse, perihilar, or unilateral GGO with or without consolidation lacking a specific distribution and are non-rounded or non-peripheral.  Few very small GGO with a non-rounded and non-peripheral distribution | “Imaging features can be seen with (COVID-19) pneumonia, though are nonspecific and can occur with a variety of infectious and noninfectious processes.” |
| Atypical appearance | Uncommonly or not reported features of COVID-19 pneumonia. | Absence of typical or indeterminate features AND  Presence of:  Isolated lobar or segmental consolidation without GGO Discrete small nodules (centrilobular, “tree- in-bud”)  Lung cavitation Smooth interlobular septal thickening with pleural effusion | “Imaging features are atypical or uncommonly reported for (COVID-19) pneumonia. Alternative diagnoses should be considered.” |
| Negative for pneumonia | No features of pneumonia | No CT features to suggest pneumonia. | “No CT findings present to indicate pneumonia. (Note: CT may be negative in the early stages of COVID-19.) |

**Table S3:** Dutch Society of Radiologists CO-RADS Reporting Structure [51]. *GGO – ground glass opacification*

| **CO-RADS** | **Findings on CT** | **Chance of COVID-19 infection** |
| --- | --- | --- |
| 1 | Normal or findings that indicate a non-infectious disease | Very Low |
| 2 | Consistent with other infections like typical bronchiolitis with tree-in-bud and thickened bronchial walls, tbc. No typical signs of COVID-19. | Low |
| 3 | Abnormalities indicating infection, but unsure whether COVID-19 is involved, like widespread bronchopneumonia, lobar pneumonia, septic emboli with ground glass opacities | Unsure or indeterminate |
| 4 | - Unilateral GCO - Multifocal consolidations without any other typical finding - Findings suspicious of COVID-19 in underlying pulmonary disease | High |
| 5 | - Multifocal GGO - Peripheral and basal distribution - Ill-defined margins - Vascular thickening - Crazy paving pattern - GGO and consolidation - reversed halo sign - Spider web appearance | Very High |
| 6 | Any finding on CT | Known COVID-19 (PCR proven) |

**Table S4:** Total severity scores (TSS) reflecting the extent of inflammatory lesions in COVID-19 [52]. Inflammatory lesions are defined as any consolidation or ground glass opacification and scored per lung lobe. The total severity score of all 5 lobes (right upper, middle, lower; left upper and lower lobes) provides a score in the range of 0 – 20.

| **Total Severity Score** | **Degree of inflammatory involvement of parenchyma** | **Description of severity** |
| --- | --- | --- |
| 0 | 0% | None |
| 1 | 1-25% | Minimal |
| 2 | 26-50% | Mild |
| 3 | 51-75% | Moderate |
| 4 | 76%-100% | Extensive |
